# Nutritional status influences peripheral immune cell phenotypes in healthy men in rural Pakistan

**DOI:** 10.1186/1742-4933-9-16

**Published:** 2012-08-03

**Authors:** Iftikhar Alam, Anis Larbi, Graham Pawelec

**Affiliations:** 1 Tübingen Ageing and Tumour Immunology Group, Zentrum für Medizinische Forschung, University of Tübingen, Waldhörnlestraße 22, D-72072, Tübingen, Germany; 2 Faculty of Agriculture, Abdul Wali Khan University Mardan, Mardan, Khyber Pakhtunkhwa (KPK), Pakistan; 3 Singapore Immunology Network (SIgN), Biopolis, Agency for Science, Technology and Research (A*STAR), Singapore, Singapore

**Keywords:** Aging, Nutrition, Immunity, T and B cells

## Abstract

Immune status is influenced by malnutrition, but how this factor interacts in developing countries and whether these differences are similar to those determined in industrialized countries, is unclear. To establish whether malnutrition-associated immune profiles in a developing country are similar to those in industrialized countries we analyzed peripheral blood immune cell phenotypes by polychromatic flow cytometry in 50 young and 50 elderly subjects. Data on anthropometrics and diet were collected through interviews. Plasma samples were analyzed for common clinical chemistry variables. Subjects in 4 BMI categories differed in their immune parameters demonstrating influence of nutritional status on immunity. This was greater within the young group and affected the CD4 subset more profoundly than the CD8 subset. No nutrition-associated differences were seen in B or NK cells. CD8+ cells as a percentage of CD3+ T cells were positively associated with plasma CRP levels but not other factors. We conclude that there are differences in the immune signatures of obese, overweight and underweight versus normal-weight young and elderly, which seem broadly similar to the more extensively-documented state reported in industrialized countries, despite the marked societal, nutritional and many other differences.

## Introduction

The effects of malnutrition on immune functions are well established as many studies in the recent past have demonstrated that age-associated malnutrition contributes to immunodeficiency [1,2]. Malnutrition is accompanied by a decrease in immunity and an increase in susceptibility to many infectious diseases. Particularly in the aged subjects, underweight or overweight and/or obesity confer increased risk of mortality [3]. Undernutrition affects many elderly not only because their nutrient intake, in general, may be inadequate [4], but also because older adults have altered requirements for several nutrients due to the effects of aging on absorption, utilization, and excretion of nutrients [5] as well as specialized nutrient needs associated with medication use, metabolic disorders, and chronic disease [6]. The effects of undernutrition on immune status may be judged from the fact that a substantial part of the lean mass of human body is comprised of lymphocytes [7]. Underweight may contribute to increased mortality caused by viral infections due to inability to meet the enhanced energy requirements associated with the immune response [8].

On the other hand, overweight and obesity are forms of malnutrition at epidemic proportions globally [1]. The comorbidities associated with obesity affect virtually every physiological system including the regulation of immunity and inflammation [2]. The relationship between obesity and immunity is logically to be expected mainly on the basis of three lines of evidence. First, obesity is linked to increased risks of virtually all types of cancers [2]. Second, obesity is closely associated with chronic, systemic inflammation, which may contribute to the development of obesity-related co-morbidity [9]. Third, a number of hormones including leptin, which is a satiety factor dysregulated in obesity, have been shown to play an important role in regulating immune functions [10]. In addition, some recent studies have shown a link between alterations in lymphocyte functions and the pathogenesis of a number of obesity-related comorbidities including atherosclerosis, steatohepatitis, and diabetes [11,12], the presence of which may underlie a number of obesity effects on the immune system.

Obese and underweight individuals may present with a difference in their immune profile as compared to people of normal weight. Our objectives were to determine the effects of malnutrition in a sample of obese and underweight young and elderly individuals in order to determine whether malnutrition has any association with alterations in the frequency of peripheral blood lymphocyte subsets. Any such study of possible associations would provide further supporting evidence for the presence of an immune diathesis of obesity and underweight, which will help to identify specific lymphocyte subsets as those most important to monitor in further studies in nutritional immunology. Furthermore, this pilot study was conducted in a rural population in a developing country in order to ascertain the general applicability of the immunological findings worldwide.

## Subjects and methods

### Study subjects

Participants in the current study were recruited from Peshawar, Pakistan. Potential subjects expressing interest in blood donation were first screened by obtaining a verbal medical history to rule out any health conditions or medication use that could affect immune response. We selected 100 subjects (50 each young and the elderly) for this study from a large number of young and elderly subjects, who were previously visited and interviewed for their anthropometric measurements and nutritional data [13]. Based on their BMI values, these subjects fell into four BMI categories, obese (N = 12), overweight (N = 12), normal weight (N = 14) and underweight (N = 12). Anthropometric measurements were carried out with the subject barefoot, wearing light clothing, and after an overnight fast. Body weight, height, and percent body fat (% BF) were measured. Body mass index (BMI) was calculated as body weight divided by the squared height (kg/m^2^). There are well-documented links between high levels of central adiposity in adults as measured by waist circumference (WC) and/or waist-to-hip ratio (WHR), and risk of obesity-related conditions including type-2 diabetes, hypertension and heart disease. These links remain even once BMI is adjusted for age, demonstrating that measures of central adiposity are independent predictors of future obesity-related diseases [14]. We, therefore, further divided our subjects into different risk categories relative to normal weight and WC. For the purpose of the present study, for WC the risk categories were defined as: low risk (LR, WC; <89 cm), moderate risk (MR, WC; 90–99 cm) and high risk (HR, WC; ≥100 cm). For WHR, the risk categories were defined as low risk (LR, WHR; < 0.89), moderate risk (MR, WHR; 0.90–0.94) and high risk (HR, WHR; ≥0.95).

### Dietary intake data

Habitual dietary intake was assessed through 24-hr Dietary Recalls (24-hr DR). Each item of food eaten during the previous 24 hrs was recalled by the subject and the amount of food eaten was used for estimation of nutrient intake using accepted food composition tables [15].

### Immunological studies

For assessment of immune signatures, blood samples were collected aseptically by venipuncture. Approximately 18 ml of whole blood (WB) was obtained from each subject, drawn into two 9 ml EDTA vacutainers (Becton Dickinson, Franklin Lakes, NJ). The samples were processed following a procedure especially developed and validated for analyses on whole frozen blood [16]. Another 10 ml blood sample was used for plasma separation for biochemical analysis. Blood and plasma samples were stored in a −80°C freezer until further analysis. After thawing in a 37°C water bath, red cells in blood samples were lysed with saline and water. The cells were then stained with 50 μl of the antibody cocktail and incubated for 30 minutes at room temperature in the dark. For intracellular FOXP3 staining, cells were resuspended in 1.0 ml FOXP3 Fix/Perm (Biolegend, San Diego), vortexed and incubated for 20 minutes at room temperature in the dark, followed by consequent washing with PFEA and FOXP3 Perm buffer. The cells were resuspended in 50 μl FOXP3 Perm buffer containing FOXP3 antibody, incubated for 30 minutes at room temperature in the dark, washed with PFEA and resuspended in PFEA. The monoclonal antibodies and fluorescent conjugates used were CD3 (Pacific Orange); CD3 (Alexa Fluor 700), CD4 (PerCP), CD8 (APC-H7), CD8 (Qdot 705); CD27 (Qdot-605), CD28 (PE), CD28 (PercP-Cy 5-5); CD45RO (Alex Flour 400); KLRG1 (Alexa Fluor 488); CD45RA (Qdot 655); CD127 (APC e-Fluor 780); CD57 (FITC); FOXP3 (PE). Cell populations were measured using an LSR-II flow cytometer and the acquisition commercial software BD FACSDiva (Becton Dickinson).

The study was approved by the Board of Studies, Department of Human Nutrition, Agriculture University Peshawar. Written informed consent was obtained from all the participants before the start of the study.

### Statistical analysis

All the data were statistically analyzed using JMP (Version 8.0. SAS, USA). As the current study involved four BMI categories, the mean values of CD4, CD8 and B cells and their subsets were taken for one-way analysis of variance (ANOVA) and post-hoc comparisons using Dunnett’s test with the normal BMI category as reference after adjusting for multiple comparison. BMI-adjusted partial correlation coefficients were calculated to establish associations between anthropometric measurements, nutrients and blood bio-chemicals with T and B cells and their subsets. To establish WC-, WHR- and % BF- associated differences in the number of CD4+, CD8+ and B cells, the percentages of these cells were compared in each of the three WC and WHR risk categories (considering low risk category as reference) and the three body fat categories (considering normal fat category as a reference). We took *p* < 0.05 to denote a significant difference.

## Results

### Age and anthropometric characteristics

Age and general characteristics of the study subjects are shown in Table 1. Age of young and the elderly subjects ranged from 18.0–29.2 (mean, SD: 24.2 ± 3.4) and 50–85.5 (mean, SD: 67.3 ± 8.7), respectively. The mean ages of the four BMI groups within young and elderly had non-significant difference (*p,* for all trends <0.05: data not shown). Compared to the elderly, the young had higher mean BMI and WHR, while the elderly had higher mean weight, WC and % BF. However, all these differences lacked statistical significance except % BF. There were significant differences in the nutrient intake between young and elderly: the young had a significantly higher intake of energy and protein (Table 1).

**Table 1:** General characteristics and nutrient intake of the subjects

**Anthropometry**	**Young**		**Elderly**		***P-value***
	**Mean (SD)**	**Range**	**Mean (SD)**	**Range**	
Age (years)	24.2 (3.43)	18.0–29.2	67.3 (8.77)	50.1–85.5	-
Weight (Kg)	67.6 (14.02)	45.3–92.4	68.7 (14.57)	46.0–97.0	0.7329
BMI (Kg/m2)	25.0 (5.37)	16.3–33.4	24.2 (5.47)	15.4–33.8	0.5056
WC	82.1 (11.20)	64.1–102.2	86.7 (12.39)	62.1–113.2	0.0735
WHR	1.0 (0.11)	0.70–1.17	0.9 (0.12)	0.67–1.21	0.3676
% BF	17.7 (8.48)	5.5–33.1	21.3 (7.99)	9.0–32.6	**0.0200**
Energy intake (Kcal)	2344 (498.8)	1262–3280	1778 (479.9)	659–2487	**<0.0001**
Protein intake (g)	48.8 (11.39)	29.5–77.2	37.6 (12.19)	14.6–68.6	**<0.0001**

Mean (STD); *p* significant at á = 0.05.

### T cell distribution in the four BMI categories of the young and elderly

When subjects were grouped according to the four BMI categories, both young and elderly showed certain differences in the percentages of CD4+ and CD8+ cells, and their subsets, as well as in B and NK cells. Tables 2 and 3 show, respectively, the percentages of CD4+ and CD8+ cells, and their subsets, according to BMI categories in the young. There were significantly lower percentages of CD4+ cells in young OB *vs.* NW. OW and UW young also tended to have lower frequencies of CD4+ cells compared to NW. The percentage of CD4+CD45RA+CD27+ cells in NW young was significantly higher than in OW. The percentages of CD4+ CD45RA-CD27- cells in OW and UW were significantly higher than in NW young (*p* for all trends < 0.05). The frequency of CD4+CD28-CD27-KLRG1+CD57+ cells in UW was also significantly higher than in NW young. The differences in the frequencies of all other subsets within CD4+cells among the three BMI groups *vs.* the NW group did not achieve statistical significance.

**Table 2:** CD4+ T cells and subsets in the 4 BMI categories of young subjects

	**BMI Category**				**p-value**		
	**NW**	**OB**	**OW**	**UW**	**OB-NW**	**OW-NW**	**UW-NW**
CD4+	37.2 (6.71)	24.3 (11.51)	24.4 (11.60)	26.8 (10.11)	**0.0014**	0.316	0.180
CD28+CD27+	54.3 (11.12)	45.3 (14.19)	45.1 (13.72)	54.1 (15.81)	0.2339	0.2213	0.2121
CD28-CD27-	12.3 (6.11)	14.1 (10.61)	14.7 (8.21)	15.0 (7.43)	0.9090	0.8150	0.7434
CD45RO+CD27-	21.2 (15.21)	17.9 (9.21)	16.1 (12.45)	22.5 (14.27)	0.8616	0.6492	0.9892
CD45RO-CD27+	27.6 (16.12)	30.9 (15.9)	31.8 (15.31)	30.3 (15.11)	0.9159	0.8439	0.4312
CD45RA+CD27+	33.6 (14.21)	27.1 (8.81)	19.6 (16.61)	21.8 (19.71)	0.5412	**0.0466**	0.1139
CD45RA-CD27-	14.1 (9.31)	36.3 (10.71)	42.1 (24.40)	54.7 (35.62)	0.3971	**0.0072**	**0.0032**
CD45RO+CD28-	8.8 (4.71)	9.5 (7.32)	6.2 (3.11)	12.6 (4.72)	0.9784	0.4521	0.1656
CD45RO-CD28+	44.4 (14.81)	31.2 (14.12)	38.1 (17.51)	30.9 (8.11)	0.5733	0.5449	0.0511
CD45RA+CD28+	33.8 (17.81)	27.1 (20.62)	17.5 (25.01)	31.3 (14.21)	0.7261	0.1053	0.1797
CD45RA-CD28-	16.7 (12.71)	19.2 (20.21)	23.4 (21.60)	23.4 (14.32)	0.9682	0.6445	0.6512
TRegs	3.9 (4.53)	5.9 (5.72)	4.8 (3.90)	3.2 (4.31)	0.5819	0.9172	0.9713
CD28+CD27+KLRG1-CD57-	37.1 (18.10)	34.6 (16.61)	30.1 (15.61)	22.4 (20.31)	0.9713	0.6355	0.1065
CD28-CD27-KLRG1+CD57+	0.6 (0.90)	0.6 (1.32)	1.0 (1.51)	2.3 (1.10)	0.5436	0.8415	**0.0028**

Significant Dunnett’s test (*p* < 0.05) comparing CD4+ T subsets with the reference group. The reference group defined as having BMI, 18.5–24.9.OB, Obese; OW, Overweight; NW, Normal Weight; UW, Underweight. The values are means (SD).

**Table 3:** CD8+ T cells and subsets in the 4 BMI categories of young subjects

	**BMI Category**				**p-value**		
	**NW**	**OB**	**OW**	**UW**	**OB-NW**	**OW-NW**	**UW-NW**
CD8+	21.2 (2.76)	17.4 (7.04)	21.5 (4.88)	19.1 (5.06)	0.1549	0.5221	0.5324
CD28+CD27+	37.4 (13.1)	32.0 (8.08)	33.4 (8.86)	32.2 (8.18)	0.3832	**0.1133**	0.4239
CD28-CD27-	29.0 (5.25)	27.0 (8.03)	28.6 (8.87)	25.6 (4.26)	0.7898	0.5432	0.4436
CD45RO+CD27-	6.4 (4.48)	8.8 (6.91)	5.7 (5.32)	7.7 (4.03)	0.5117	0.7566	0.8489
CD45RO-CD27+	47.9 (7.74)	43.2 (10.23)	45.0 (13.11)	32.6 (7.86)	0.4886	0.8057	**0.0008**
CD45RA+CD27+	32.8 (12.31)	29.8 (11.89)	32.3 (9.0)	28.7 (13.77)	0.8651	0.8774	0.7262
CD45RA-CD27-	10.4 (9.18)	15.5 (9.40)	12.7 (9.36)	15.3 (10.98)	0.4219	0.8945	0.4625
CD45RO+CD28-	11.4 (6.80)	14.1 (5.57)	17.3 (5.46)	12.5 (4.90)	0.5072	**0.0360**	0.9321
CD45RO-CD28+	44.1 (13.06)	43.0 (16.10)	35.7 (10.53)	38.7 (11.45)	0.6588	0.2479	0.5923
CD45RA+CD28+	41.9 (14.50)	43.5 (17.43)	46.2 (17.45)	45.4 (15.10)	0.6466	0.8344	0.9074
CD45RA-CD28-	20.1 (4.80)	17.1 (5.54)	20.5 (8.17)	17.1 (9.24)	0.5764	0.6455	0.5887
CD28+CD27+KLRG1-CD57-	24.3 (22.76)	20.9 (15.45)	22.0 (19.53)	12.8 (12.42)	0.3883	0.4239	**0.0133**
CD28-CD27-KLRG1+CD57+	1.0 (1.45)	1.0 (1.71)	2.4 (1.32)	3.4 (1.41)	0.7898	0.5468	0.0487

Significant Dunnett’s test (*p* < 0.05) comparing CD8+ T subsets with the reference group. The reference group defined as having BMI, 18.5–24.9. OB, Obese; OW, Overweight; NW, Normal Weight; UW, Underweight. The values are means (SD).

Regarding CD8+CD45RO-CD27+ cells, the difference between UW *vs.* NW young was highly significant. OW young had a higher frequency of CD4+CD45RO+CD28- cells compared to NW young and UW young had significantly lower CD8+CD28+CD27+KLRG1-CD57- cells than NW young. There tended to be some further differences in the frequencies of other subsets within CD8+ cells among the three BMI groups *vs.* NW group of young, but none of these reached significance (*p* for all trends ≥0.05).

Tables 4 and 5, respectively, show the frequencies of CD4+ cells, CD8+ cells, and their subsets in the four BMI categories of the elderly. The frequencies of all other subsets within CD4+ cells among the three BMI categories *vs.* NW did not differ significantly except for CD45RA+CD27+, CD45RA-CD27-, and CD45RO-CD28+ cells. The percentage of CD45RA+CD27+ cells in OB was significantly higher than NW. Similarly, CD4+CD45RO-CD28+ cells in OB were higher than in NW. The percentage of CD8+ cells in UW elderly was significantly lower than in NW. None of the other differences in the frequencies of subsets within CD8+ cells among these three BMI categories *vs.* NW achieved significance.

**Table 4:** CD4+ T cells and subsets in the 4 BMI categories of elderly subjects

	**BMI Category**				**p-value**		
	**NW**	**OB**	**OW**	**UW**	**OB-NW**	**OW-NW**	**UW-NW**
CD4+	26.9 (12.21)	23.8 (9.67)	26.2 (9.91)	22.5 (7.35)	0.7925	0.5421	0.6352
CD28+CD27+	17.9 (10.04)	13.0 (9.87)	16.6 (10.03)	48.1 (14.3)	0.4205	0.9944	0.4745
CD28-CD27-	43.2 (12.73)	45.3 (16.01)	47.4 (9.37)	39.3 (12.45)	0.9611	0.7724	0.6809
CD45RO+CD27-	39.3 (10.46)	31.6 (12.75)	31.0 (17.88)	22.5 (14.21)	0.5734	0.2912	0.5154
CD45RO-CD27+	13.3 (9.36)	22.1 (13.02)	20.5 (15.92)	30.3 (15.19)	0.1882	0.3334	0.9878
CD45RA+CD27+	14.0 (10.41)	26.3 (14.89)	19.9 (10.98)	15.7 (12.71)	**0.0303**	0.4581	0.988
CD45RA-CD27-	56.2 (19.32)	33.6 (21.56)	45.6 (17.47)	49.5 (26.31)	**0.0269**	0.4559	0.7671
CD45RO+CD28-	29.7 (12.89)	20.5 (9.97)	21.3 (14.16)	27.4 (17.8)	0.2348	0.3035	0.7543
CD45RO-CD28+	16.6 (13.32)	33.3 (13.28)	21.0 (13.58)	21.4 (14-8)	**0.0093**	0.7072	0.7521
CD45RA+CD28+	16.0 (12.23)	19.2 (10.49)	13.3 (11.20)	14.8 (12.8)	0.8368	0.8951	0.3426
CD45RA-CD28-	47.2 (16.28)	36.8 (16.17)	40.9 (13.92)	48.6 (14.31)	0.2274	0.6194	0.5461
Tregs	9.4 (4.81)	7.9 (4.24)	9.0 (4.66)	14.0 (7.67)	0.8512	0.4776	0.0918
CD28+CD27+KLRG1-CD57-	8.5 (8.60)	8.3 (6.73)	7.9 (5.71)	13.2 (10.36)	0.5123	0.4521	0.3249
CD28-CD27-KLRG1+CD57+	10.7 (4.60)	10.4 (4.67)	9.8 (3.57)	13.0 (7-08)	0.5412	0.3421	0.4512

Significant Dunnett’s test (*p* < 0.05) comparing CD4+ and subsets with the reference group. The reference group defined as having BMI, 18.5–24.9. OB, Obese; OW, Overweight; NW, Normal Weight; UW, Underweight. The values are means (SD).

**Table 5:** CD8+ T cells and subsets in the 4 BMI categories of elderly subjects

	**BMI Category**				**p-value**		
	**NW**	**OB**	**OW**	**UW**	**OB-NW**	**OW-NW**	**UW-NW**
CD8+	28.5 (4.8)	26.9 (5.8)	25.0 (7.31)	22.5 (6.31)	0.8523	0.3514	0.0443
CD28+CD27+	31.4 (7.32)	27.9 (11.42)	34.6 (6.42)	32.7 (6.68)	0.6545	0.5134	0.4219
CD28-CD27-	12.1 (6.52)	14.4 (6.63)	11.9 (6.01)	8.4 (4.42)	0.5696	0.648	0.9613
CD45RO+CD27-	15.1 (11.21)	9.7 (5.86)	9.2 (5.21)	12.1 (10.01)	0.1541	0.7298	0.5421
CD45RO-CD27+	36.9 (7.21)	34.2 (8.31)	38.9 (9.61)	35.2 (13.61)	0.3854	0.5975	0.8712
CD45RA+CD27+	18.3 (7.31)	16.0 (9.02)	17.8 (11.31)	14.7 (7.91)	0.3043	0.4237	0.6577
CD45RA-CD27-	44.9 (9.31)	46.8 (13.01)	40.9 (12.61)	48.9 (14.23)	0.3175	0.8401	0.2411
CD45RO+CD28-	28.8 (10.01)	35.2 (6.71)	34.3 (14.91)	30.3 (9.01)	0.8421	0.5411	0.6240
CD45RO-CD28+	22.1 (8.0)	17.2 (7.71)	19.9 (9.21)	19.9 (8.31)	0.4954	0.1544	0.7662
CD45RA+CD28+	11.2 (8.61)	12.5 (7.21)	13.9 (9.91)	10.9 (5.81)	0.4954	0.1544	0.7662
CD45RA-CD28-	51.0 (13.91)	59.5 (12.71)	57.4 (19.21)	54.8 (15.61)	0.2732	0.2153	0.7001
CD28+CD27+KLRG1-CD57-	10.5 (9.51)	9.4 (4.31)	8.3 (5.21)	7.9 (7.01)	0.8250	0.1931	0.5442
CD28-CD27-KLRG1+CD57+	9.0 (6.11)	6.5 (2.11)	5.3 (2.21)	9.6 (8.31)	0.6545	0.5134	0.2775

Significant Dunnett’s test (*p* < 0.05) comparing CD8+ and subsets with the reference group. The reference group defined as having BMI, 18.5–24.9. OB, Obese; OW, Overweight; NW, Normal Weight; UW, Underweight. The values are means (SD).

### B cell and NK cell distribution in the four BMI categories

Tables 6 and 7, respectively, show a comparison of the percentages of B cells and their subsets (IgD+CD27-, IgD-CD27+), and NK cells (CD56+CD16+), among the three BMI categories *vs.* the NW category of young and elderly. There were no significant differences in either of these phenotypes in the young. In the elderly, however, significant differences in the percentages of IgD+CD27- cells were apparent between UW and NW; and in IgD-CD27+ cells between UW *vs.* NW; and finally, in NK cells between NW and OB.

**Table 6:** B cells and NK cells in the 4 BMI categories of young subjects

	**BMI Category**				**p-value**		
	**NW**	**OB**	**OW**	**UW**	**OB-NW**	**OW-NW**	**UW-NW**
B cells	2.9 (2.45)	3.8 (2.44)	3.0 (2.10)	1.9 (1.80)	0.6368	0.5421	0.5520
IgD+CD27-	39.5 (13.0)	39.9 (11.27)	30.2 (11.35)	32.2 (16.20)	0.8581	0.1896	0.3731
IgD-CD27+	16.3 (14.80)	18.3 (12.11)	22.9 (7.11)	16.8 (13.82)	0.7645	0.3172	0.8066
NK Cells	1.0 (3.05)	0.6 (0.71)	0.5 (0.64)	0.5 (1.36)	0.331	0.8060	0.7704

Significant Dunnett’s test (*p* < 0.05) comparing B cells and NK cells with the reference group. The reference group defined as having BMI, 18.5–24.9. OB, Obese; OW, Overweight; NW, Normal Weight; UW, Underweight.The values are means (SD).

**Table 7:** B cells and NK cells in the 4 BMI categories of elderly subjects

	**BMI Category**				**p-value**		
	**NW**	**OB**	**OW**	**UW**	**OB-NW**	**OW-NW**	**UW-NW**
B cells	3.8 (2.19)	4.3 (2.24)	4.0 (1.57)	2.7 (1.81)	0.8483	0.8128	0.3471
IgD+CD27-	14.5 (6.14)	7.8 (6.68)	5.4 (4.35)	25.1 (8.12)	0.4307	0.2002	**0.0159**
IgD-CD27+	24.6 (16.33)	29.9 (12.8)	34.5 (11.21)	12.4 (13.08)	0.6357	0.1720	**0.042**
NK Cells	2.0 (2.2)	0.7 (1.16)	1.0 (1.59)	1.0 (1.70)	**0.0488**	0.3822	0.3614

Significant Dunnett’s test (*p* < 0.05) comparing B cells and NK cells with the reference group. The reference group defined as having BMI, 18.5–24.9. OB, Obese; OW, Overweight; NW, Normal Weight; UW, Underweight. The values are means (SD).

### T and B cell distribution in WCC, WHR risk categories

Table 8 shows a comparison of the mean percentages of CD4+, CD8+ and B cells in the three WC risk categories (i.e. HR, MR and LR) of young and elderly. For young subjects, CD8+ and B cells, respectively, in HR-WC and MR-WC groups differed significantly from those in the LR-WC group (the control group for comparison). However, no significant differences were observed either in CD4+ cells, CD8+ cells or B cells between the two risk groups (HR-WC, MR-WC) and the control group (LR-WC) in the elderly. Similarly, there were no significant differences in the mean percentages of CD4+ cells, CD8+cells or B cells for the three WHR categories of young and elderly. Young low fat (LF) subjects had significantly lower CD4+ cells compared to normal fat (NF) subjects.

**Table 8:** Multiple comparisons between the low risk group and other groups for percent CD4+, CD8+ and B cells

	**Cell type**	**WC (Cat)**	**Mean Diff. (LR-cat)**	***p*value**^**1**^	**WHR (Cat)**	**Mean Diff. (LR-cat)**	***p*value**^**1**^	**BF (cat)**	**Mean Diff. (NF-cat)**	***p*value**^**1**^
**Young**	CD4+	MR	−15.1	0.070	MR	−7.84	0.172	HF	−1.47	0.113
		HR	−0.93	0.738	HR	−0.64	0.078	LF	1.24	**0.020**
	CD8+	MR	−4.11	0.346	MR	−3.04	0.990	HF	−1.11	0.195
		HR	0.35	**0.014**	HR	−4.57	0.632	LF	−1.92	0.343
	B cells	MR	−1.0	0.944	MR	−0.92	0.434	HF	−1.45	0.943
		HR	0.41	**0.006**	HR	−1.04	0.969	LF	−0.72	0.281
**Elderly**	CD4+	MR	−9.75	0.983	MR	−5.78	0.912	HF	−6.80	0.489
		HR	−7.67	0.727	HR	−6.46	0.844	LF	−7.16	0.550
	CD8+	MR	−3.62	0.822	MR	−6.41	0.125	HF	−4.03	0.932
		HR	−6.39	0.919	HR	−2.41	0.484	LF	−2.56	0.242
	B cells	MR	−2.18	0.793	MR	−1.46	0.921	HF	−1.02	0.707
		HR	−2.07	0.873	HR	−2.23	0.421	LF	−0.77	0.226

^1^The mean difference is significant against low risk (LR) subjects at á = 0.05 level; MR, moderate risk; HR, high risk. For % BF, the mean difference is significant against normal fat subjects at the 0.05 level; LF, low; HF, high fat; Cat, category; ^1^p-values were calculated using Dunnett’s test. Positive values of the mean difference show pairs of means that are significantly different.

### Correlation between age, anthropometrics and CD8+, CD4+ cells and subsets

**Figure 1:**
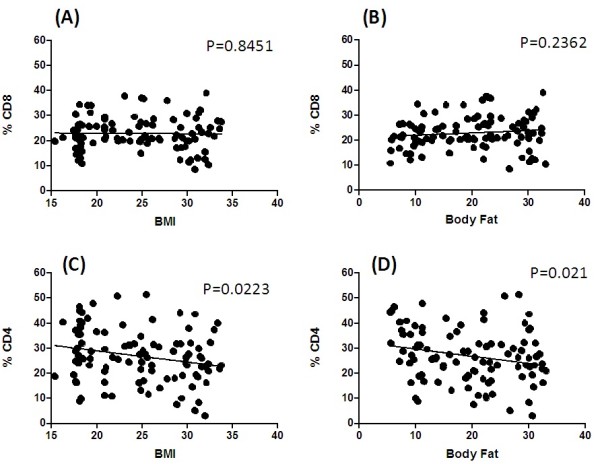
Correlations between percentages of CD8+ and CD4+ cells and BMI and body fat. Significant at p < 0.05.

Figure 1 depicts correlation analyses. While BMI and % BF had no significant effect on the percentage of CD8+ T cells (*p* for all trends ≥ 0.05) (Figure. 1 A, B), these values were inversely correlated with CD4+ cells (Figure 1 C, D). None of the nutrient values assessed here correlated significantly with either CD8+ or CD4+ T cells or their subsets (*p* for all trends ≥ 0.05). There was a significant increase in both CD8+ and CD4+ cells with an increase in plasma CRP. The other plasma factors (albumin, total protein, triglycerides, and ferritin) had no significant correlations with the percentages of CD8+ and CD4+ cells.

## Discussion

The effects of nutrition on immune functions have been extensively investigated, but almost exclusively in so-called “WEIRD” subjects (Western, educated, industrialized, rich, and democratic) and rarely in concert. It is not established whether immune alterations found in these populations are representative of the majority of the world’s peoples. The possible detrimental effects of aging on the immune system are further aggravated by numerous physiological and physical conditions, which in many cases can be associated with malnutrition. In the present study, we investigated the effects of nutritional status on immune signatures in a group of healthy young and elderly individuals from a rural area in a developing country where nutritional issues tend to be even more extreme than in developed countries (both in terms of over-as well as under-weight).

The primary findings regarding relationships between nutritional status and immune parameters investigated in this study were that there were some significant differences in the subsets of CD4+ and CD8+ cells but only tendential differences in percentages of B cells and NK cells between young and elderly men based on their nutritional status. While the current study observed changes in both CD4+ and CD8+ compartments, the impact of nutritional status on CD4+ cells and their subsets was more profound than on CD8+ cells and subsets (Tables 2,3,4 and 5). Others have previously suggested that fat (one of the predictors of nutritional status) has a more direct effect on CD4+ T cell count, total lymphocyte count, and WBC count than on CD8+ T cells [17]. CD4+ subtypes are directly stimulated by various cytokines including tumor necrosis factor-α, which contributes to the differentiation of CD4+ T cells into the TH1 subset [18] and leptin. In contrast, cytokines produced by fat tissue are not central to CD8+ activation. Therefore, whereas fat directly influences CD4+ cell counts via the action of various adipokines, it may influence CD8+ counts only indirectly via its ability to activate CD4+ T cells.

In contrast to what we initially expected, the current study did not identify many differences among the three BMI categories versus normal. Nevertheless, our findings on the comparison between OB and NW subjects are in agreement with Lynch *et al.*[19], who reported significantly more CD8+ and NK cells in lean controls compared to obese individuals. They further reported no differences in CD4+ levels between obese and lean individuals. In addition, they observed that the phenotypes of immune cells were also different between obese and lean individuals with regard to expression of activation markers and that obese individuals expressed significantly less CD45RA on their T cells. However, when obese individuals were further divided into metabolically healthy (MH) and unhealthy (UH) groups, it was found that circulating NK cells and CD8+ T cell levels were significantly reduced only in the UH obese group. In our study, all the overweight and obese individuals seem more likely to be metabolically healthy as our inclusion criteria excluded all those who had a present or recent past history of diseases including diabetes, hypertension, CVD etc. We, therefore, need further studies in elderly Pakistani subjects including both metabolically healthy and unhealthy obese elderly individuals to investigate the differences as reported previously [20]. It has been suggested that the unique metabolically healthy subgroup of obese individuals appear to be protected or more resistant to the development of comorbidities associated with obesity [20]. Despite having excessive body fat, these individuals display a favorable metabolic profile characterized by high levels of insulin sensitivity, no hypertension, normal lipid, inflammation, and hormonal profiles and, importantly in the context of the present study, a favorable immune profile [19].

In the present study, young and elderly subjects were separated into high, medium and low risk categories on the basis of their WC and WHR values. The comparisons between the low risk (LR) and the other risk groups (high risk, HR; medium risk, MR) show that HR-WC young subjects had significantly (*p* = 0.0145) lower percentages of CD8+ cells (*p =* 0.014) and B cells (*p* = 0.0201) compared to the LR-WC category. In the elderly subjects, there were also differences between these categories but none of them was significant (*p* for all trends ≥0.05). In the present study, the high body fat (HF-% BF) category in young had significantly (*p* = 0.0201) lower percentages of CD4+ cells compared to the normal fat (NF) category. In general, it has been shown that a decrease in the lean body mass is related to the decrease in body cell mass [21] and that body cell mass depletion is out of proportion to loss of body weight for fat [22]. Furthermore, these results may suggest that compared to the elderly, in young people, WC, WHR and % BF are more sensitive anthropometric measurements influencing the percentages of circulating CD4+, CD8+ and B cells. We are not aware of concrete evidence from previous studies to support the present observations. Some conflicting results, however, demonstrated an increase in CD4+ cells and a decrease in CD8+ cells in obese people (based on BMI) [9]. Others have attempted similar studies of lymphocyte subset frequency in obesity, with conflicting results. Total circulating lymphocytes and monocytes were reported as increased [23] or the same [24] as in the lean controls. Lymphocyte subsets have also been studied, and some investigators have found no differences in numbers of circulating T-cells, B cells, and NK cells [25], while others have shown increased or decreased lymphocytes and T-cells in obese people, and correlated the magnitude of these differences with increasing BMI [26]. Still other investigators have demonstrated a relationship between morbid obesity and CD8+ count only, but not mere overweight and/or obesity when compared with normal weight [23]. Despite these conflicting results, a preponderance of evidence suggests alterations in the immune system of both underweight and obese individuals. For example, monocyte function has been shown to alter in obese humans resulting in increased oxidative burst and phagocytic activity [23]. Such alterations in monocyte function could contribute to the state of systemic inflammation associated with obesity. T-cell phenotypes are likewise altered in obesity as reported previously [23].

No significant differences were found in the four BMI categories of young and the elderly regarding the number of B cells (Tables 6 and 7). In the IgD+CD27-, IgD-CD27+ and NK cells (CD16/CD56) significant differences were noted, respectively, between UW vs. NW; UW vs. NW and OB vs. NW elderly (Table 7). Previous research concerning differences in these cellular phenotypes in the well- and malnourished elderly is scarce. Some studies on the relationship between weight and NK cell number in other age groups, however, have shown a close but conflicting link between body weight and NK cells. Kelley *et al.*[27] reported that individuals who reported losing weight had fewer and less effective NK cells than those who had never lost weight. Likewise, Scanga and co-workers [28] pointed out that obese women consuming a restricted diet had apparent decrements in NK cell cytotoxicity. However, another experimental trial by concerning the influence of obesity on immune response in an adult population indicates that obesity is related to lower T and B-cell mitogen-induced lymphocyte proliferation but normal numbers and function of NK cells [23], while Lynch *et al.,* have shown decreased NK cell levels only in metabolically unhealthy obese compared to a similarly obese but metabolically healthy group [19]. This study had also shown that the levels and phenotypes of NK cells in metabolically healthy obese were similar to lean healthy controls. This decrease in number of NK cells in the unhealthy obese suggests that the immune system is altered in obese people who are at risk from obesity-related comorbidities. In children, a number of studies demonstrated lower proportions of B cells in malnourished compared to well-nourished individuals (nutritional status assessed by weight) [29]. Similarly, a study on overweight children found elevated counts in most types of circulating immune cells, including CD19+ B Lymphocytes, suggesting the presence of low-grade systemic inflammation [30]. In contrast, some other researchers have reported no differences between the percentages of B lymphocytes in malnourished versus well-nourished subjects [31]. If obesity is considered an inflammatory disease [32] then one might expect that NK cell number, or activity, should be increased. In contrast, results on adults suggest that NK cell number and activity may not be changed by obesity [23]. Thus, our results (Table 6) showing no significant difference in NK cells between OB and NW young are in agreement with these previous findings. Taken together, all these studies confirm that malnutrition (whether obesity or underweight) has an effect on the circulating B and NK cells, particularly in the elderly.

In the present study (Figure 1 C D), BMI and % BF were inversely correlated with percent CD4+ cells (*p* = 0.0223; *p =* 0.021, respectively), while none of the nutrients (including energy intake) correlated significantly with either CD8+ or CD4+ cells. Although energy intake has been shown to have some effects on the number of circulating lymphocytes, results are disparate. For example, some studies have shown that low energy intake is associated with a reduction in lymphocyte number and proliferation [33], while others have shown no effect of energy on the number of lymphocytes [34,35]. Similarly, except for plasma CRP, none of the plasma factors measured (albumin, total protein, triglycerides, and ferritin) had a significant relationship with either percent CD8+ or CD4+ cells, although a positive correlation between serum albumin and CD4 count and between triglycerides, CD4 and CD8 counts has been reported in a previous study with a group of dialysis patients [36]. The same study also reported non-significant correlations between CD4, CD8 and BMI, as shown in another recent study in Korean elderly [34]. Yet another study [37] demonstrated serum albumin concentrations correlated positively with some lymphocyte sub-populations.

### Strengths and limitations of the current study

This is a pilot study with relatively few individuals and small group sizes; many of the tendencies noted here could likely achieve statistical significance in larger groups. This needs exploring. Potential gender differences could not be addressed because only men could be accessed for this study, but comparisons with women need to be made. We used frozen whole blood rather than isolated PBMC for enumeration of immune cells after careful validation of this method before actual analysis [16]. Other major limitations of this study include a lack of data on immune functions; however, this was outside the scope of this pilot study. Thus, although percentages of certain immune cells were associated with weight, BMI and body fat, we could not evaluate potential differences in the functionality of these cells, but hypothesize that they are present. This remains to be tested.

## Conclusions

It is evident from the results of the current study that a state of malnutrition that is nonetheless not overtly associated with diseases such as diabetes, arthritis, and cardiovascular diseases, may still markedly affect cells of the immune system of otherwise healthy young and elderly men. Additionally, future studies will need to measure the activity of immune system cells as well cell counts, because increases or decreases in cell numbers may not necessarily mean a higher or lower associated activity. Even with its limitations, a striking finding of this study was that despite the great differences between the rural Pakistani population tested here and the commonly-reported studies on “WEIRD” subjects in the literature, the overall differences in immune profiles between younger and older people were generally very similar.

## Competing interests

All authors declare no competing interests.

## Authors’ contribution

IA wrote the manuscript. All authors edited the paper and approved its final version.
